# Supplementary material for: Diabetes downregulates the antimicrobial peptide psoriasin and increases E. coli burden in the urinary bladder
Source: Nat Commun. 2022 Sep 20;13:4983. doi: 10.1038/s41467-022-32636-y (PMC9489794; doi:10.1038/s41467-022-32636-y)
Supplement: Supplementary file 1 — Supplementary Information [file 41467_2022_32636_MOESM1_ESM.pdf]

Supplementary file

Diabetes downregulates the antimicrobial peptide psoriasin and increases *E. coli* burden in the urinary bladder.

Soumitra Mohanty[1,2], Witchuda Kamolvit[1,2], Andrea Scheffschick[3], Anneli Björklund[4,5], Jonas Tovi[6], Alexander Espinosa[3], Kerstin Brismar[5], Thomas Nyström[7], Jens M. Schröder[8], Claes-Göran Östenson[5], Pontus Aspenström[9], Hanna Brauner[3, 10], Annelie Brauner *[1,2].

[1] Department of Microbiology, Tumor and Cell Biology, Karolinska Institutet, Stockholm, Sweden.

[2] Division of Clinical Microbiology, Karolinska University Hospital, Stockholm, Sweden.

[3] Department of Medicine, Solna, Stockholm, Sweden

[4] Center for Diabetes, Academic Specialist Center, Stockholm County Council, Sweden

[5] Department of Molecular Medicine and Surgery, Karolinska Institutet, Stockholm, Sweden

[6] Capio Health Care Center, Solna, Sweden

[7] Department of Clinical Science and Education, Division of Internal Medicine, Unit for Diabetes Research, Karolinska Institute, South Hospital, Stockholm, Sweden.

[8] Department of Dermatology, Venerology and Allergology, University Hospital Schleswig-Holstein, Kiel, Germany

[9] Rudbeck Laboratory, Department of Immunology, Genetics and Pathology (IGP), Uppsala University, Uppsala, Sweden

[10] Dermato-Venereology Clinic, Karolinska University Hospital, Stockholm, Sweden.

* Corresponding author: Annelie Brauner, Department of Microbiology, Tumour and Cell Biology, Division of Clinical Microbiology, Karolinska Institutet and Karolinska University Hospital, 17176 Stockholm, Sweden; Phone +46 8 51770000, Fax: +46 8 308099. E-mail: Annelie.Brauner@ki.se

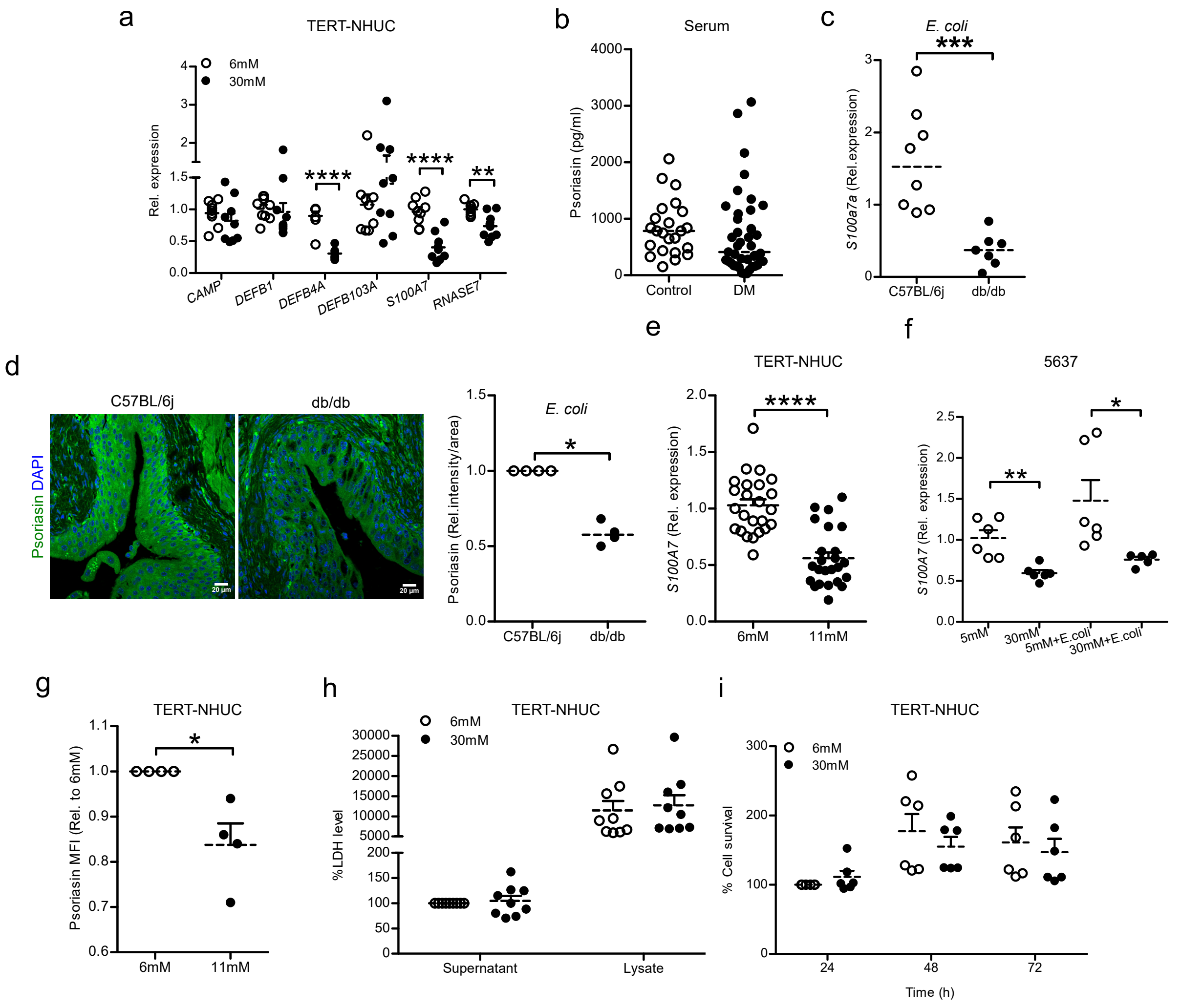

Fig. S1. Effect of high glucose on psoriasin and cell viability. (a) Expression of *CAMP* (n=9), *DEFB1* (n=9), *DEFB4A* (6mM: n=8; 30mM: n=6), *DEFB103A* (n=9), *S100A7* (n=9) and *RNASE7* (n=9) mRNA after 24 h treatment with glucose (normal=6mM: high=30mM) in TERT-NHUC cells (unpaired two-tailed t test, *p*=0.0020, *p*≤ 0.0001). **(b)** Serum psoriasin levels of patients with diabetes (DM) (n=41) and non-diabetic individuals (n=23) (unpaired two-tailed t test). **(c)** Expression of *S100a7a* mRNA in diabetic, db/db, (n=7) and non-diabetic, C57BL/6j (n=8) mouse urinary bladders after 7 days *E. coli* infection (unpaired two-tailed t test, *p*=0.0007). **(d)** Representative psoriasin staining of sections from mouse bladders after 7 days *E. coli* infection (n=4 each) (Mann-Whitney two-tailed test, *p*=0.0211). **(e)** Expression of *S100A7* mRNA in TERT-NHUC after 24 h exposure to high glucose (n=24) (unpaired two-tailed t test, *p*≤0.0001). **(f)** Expression of *S100A7* mRNA after 15 mins *E. coli* infection at MOI 10 in 5637, uroepithelial cells 24 h prior treatment with low and high glucose (n=6; 30mM+*E.coli*: n=5) (unpaired two-tailed t test, *p*=0.0306, *p*=0.0020). **(g)** Flowcytometric analysis of intracellular psoriasin (mean fluorescence intensity, MFI) in high glucose treated TERT-NHUC cells after 36 h (n=4) (Mann-Whitney two-tailed test, *p*=0.0211). **(h)** LDH levels in supernatant and cell lysates of TERT-NHUC cells treated with high glucose for 24h (n=9) (One-way ANOVA). **(i)** Metabolic activity and cell proliferation analyzed with XTT assay in high glucose treated TERT-NHUC cells at 24, 48 and 72 h (n=6) (One-way ANOVA). *In vitro* experiments were performed in duplicate or triplicate with at least 3 independent experiments and presented as mean + SEM, statistical outliers defined by Grubb's test were excluded. For *in vivo* and human material analysis, individual values and median are shown, *p < 0.05 **p < 0.01, ***p < 0.001 and ****p < 0.0001. Source data are provided as a source data file.

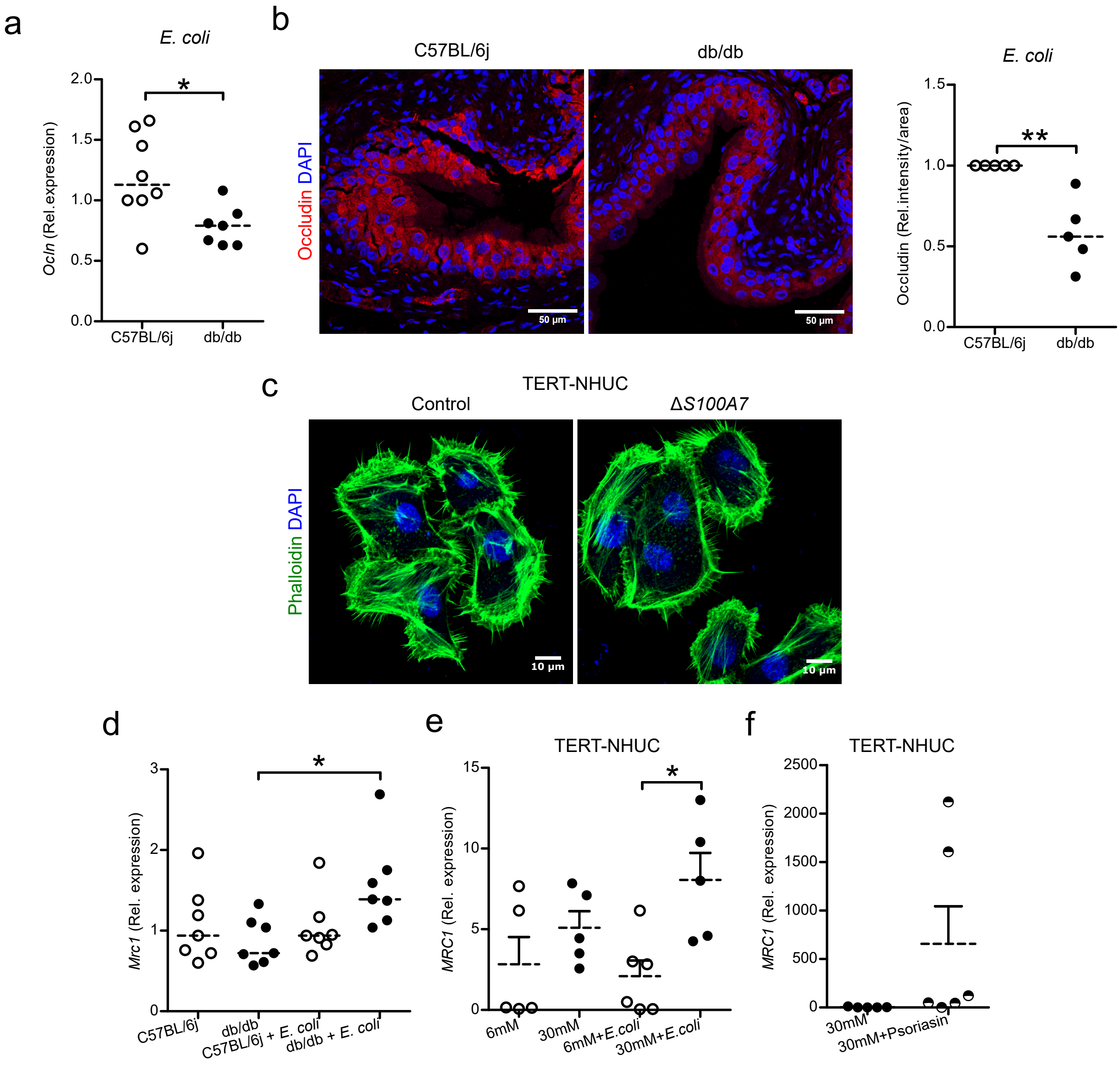

Fig. S2. High glucose and psoriasin effect on occludin and MRC1 expression. (a) Expression of *Ocln* mRNA in diabetic, db/db (n=7) and non-diabetic, C57BL/6j (n=8) mouse urinary bladders after 7 days *E. coli* infection (unpaired two-tailed t test, *p*=0.0155). **(b)** Occludin staining of sections from mouse bladders in db/db and C57BL/6j (n=5 each) after 7 days *E. coli* infection (Mann-Whitney two-tailed test, *p*=0.0075). **(c)** Representative microscopic analysis of phalloidin and nucleus in control and psoriasin deleted TERT-NHUC cells (n=12). **(d)** Expression of *Mrc1* mRNA in urinary bladders of 24 h *E. coli* infected non-diabetic, C57BL/6j and diabetic, db/db mice (n=7), PBS treated mice served as uninfected control (One-way ANOVA, multiple comparison, *p*≤0.05). **(e)** *MRC1* mRNA analysis after 24 h of glucose (normal=6mM: high=30mM) treatment in TERT-NHUC cells (n=5; 6mM +*E.coli*: n=6), followed by 1 h infection with *E. coli* (One-way ANOVA, multiple comparison, *p*≤0.05). **(f)** *MRC1* mRNA after 24 h in high glucose and psoriasin (1600nM) peptide treated TERT-NHUC (30mM: n=5; 30mM +Psoriasin: n=6) (unpaired two-tailed t test). *In vitro* experiments were performed in duplicate or triplicate with at least 3 independent experiments and presented as mean + SEM, statistical outliers defined by Grubb's test were excluded. For *in vivo* analysis, individual values and median are shown, **p* < 0.05 and ***p* < 0.01. Source data are provided as a source data file.

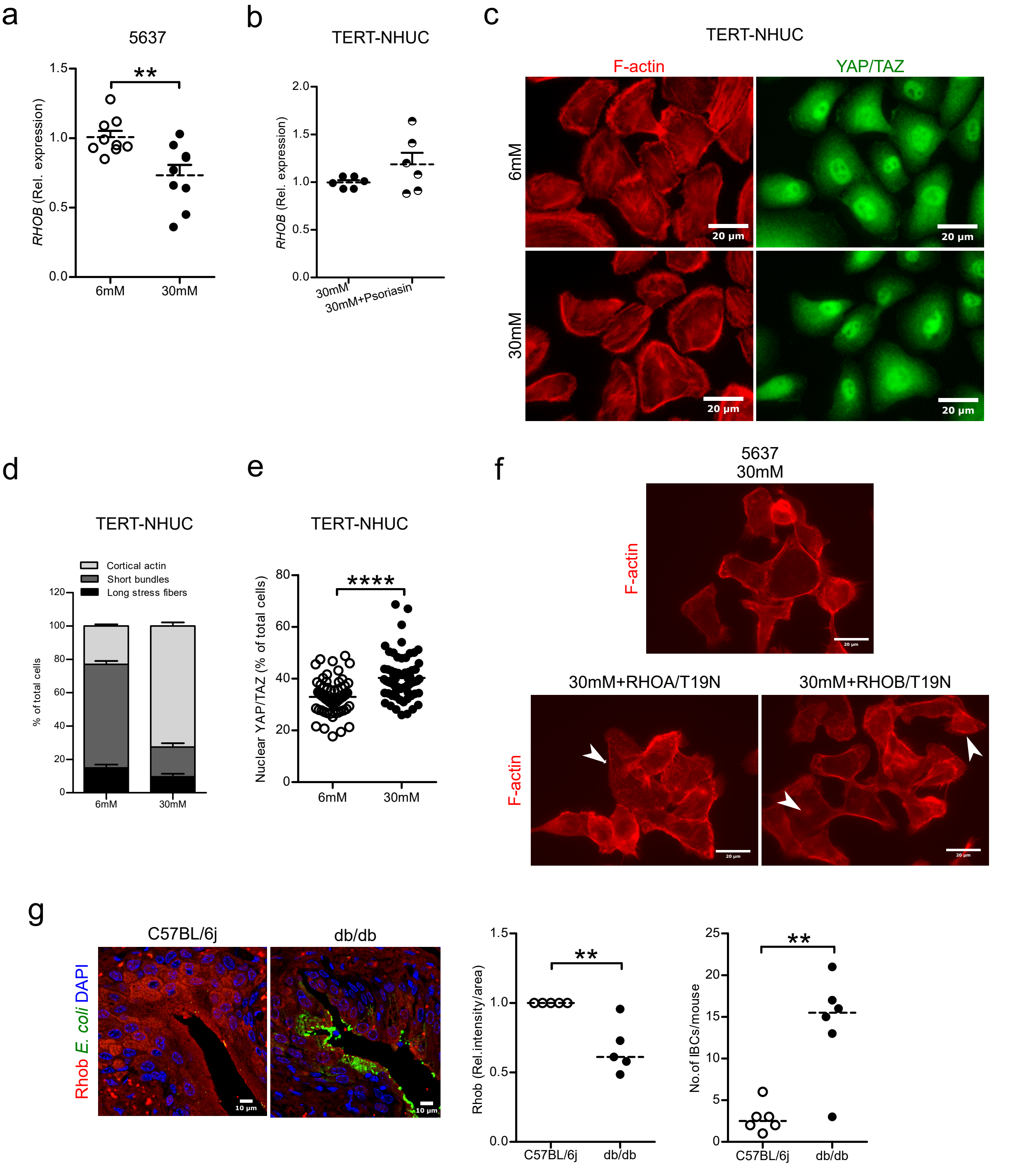

Fig. S3. Impact of high glucose and *E. coli* infection on the cytoskeleton (a) *RHOB* mRNA in glucose (normal=6mM: high=30mM) treated human uroepithelial cells, 5637 after 24 h (n=9) (unpaired two-tailed t test, *p*=0.0060). **(b)** *RHOB* mRNA after 24 h of high glucose treatment without and with psoriasin (1600nM) peptide in human uroepithelial cells TERT-NHUC (n=6) (unpaired t test, two-tailed). **(c)** Representative image depicting F-actin and YAP/TAZ in TERT-NHUC treated high glucose for 24 h (6mM, n=284; 30mM, n=248). **(d)** F-actin filaments (6mM, n=284; 30mM, n=248), (One-way ANOVA, multiple comparison) and **(e)** nuclear YAP/TAZ (6mM, n=68; 30mM, n=65) (unpaired two-tailed t test, $p \leq 0.0001$) were quantified in both normal and high glucose (6mM, n=284; 30mM, n=248). **(f)** Representative image of F-actin in high glucose treated, RHOA/B T19N transfected 5637 cells (marked with arrowhead), (30mM, n=61; 30mM+RHOA/T19N, n=41; 30mM+RHOB/T19N, n=30). **(g)** Representative sections from mouse bladders 24 h post infection, stained for Rhob and *E. coli* in non-diabetic, C57BL/6j and diabetic mice (n=5 each). Expression of Rhob detected in the superficial umbrella cells (Mann-Whitney two-tailed test, *p*=0.0075), relative number of IBCs in each mouse (n=6 each) (unpaired two-tailed test, *p*=0.0013). *In vitro* experiments were performed in duplicate or triplicate with at least 3 independent experiments and presented as mean + SEM, statistical outliers defined by Grubb's test were excluded. For *in vivo* analysis individual values and median are shown, ***p*<0.01 and *****p*<0.0001. Source data are provided as a source data file.

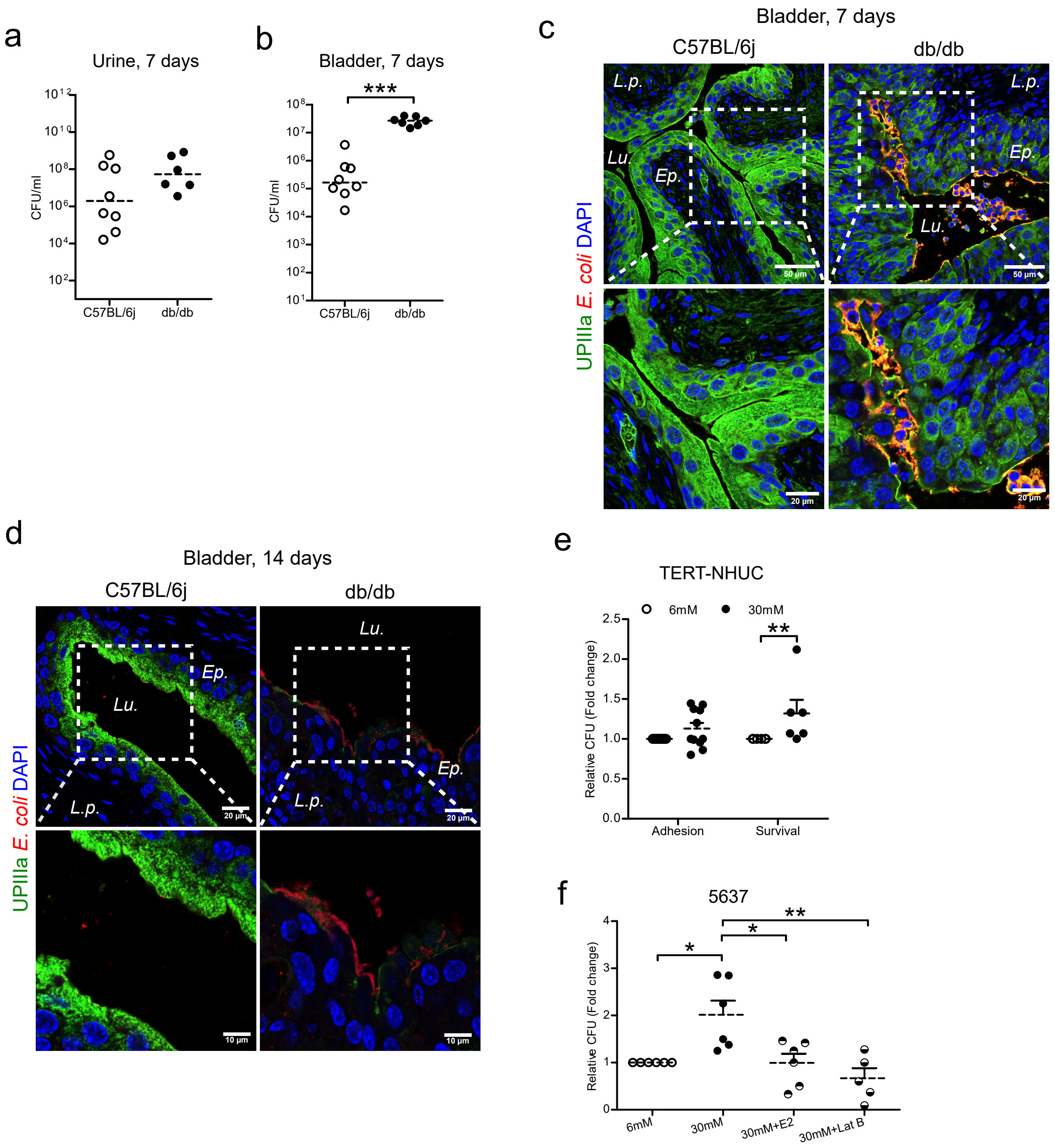

Fig. S4. The impact of high glucose on bacterial clearance. Total bacterial count was determined in **(a)** urine and **(b)** urinary bladders of non-diabetic, C57BL/6j (n=8,8) and diabetic, db/db (n=6,7) mice respectively after 7 days of *E. coli* infection (unpaired two-tailed t test, $p≤0,0001$). Representative sections from mouse bladders infected for **(c)** 7 and **(d)** 14 days were stained for UPIIIa and *E. coli* in C57BL/6j and db/db mice (n =6 each). *L.p.*, lamina propria; *Lu.*, lumen; *Ep.*, epithelium. **(e)** Adhesion (n=11) and survival assays (n=6) were performed in human uroepithelial cells, TERT-NHUC after prior treatment with glucose (normal=6mM: high=30mM) for 24 h, followed by *E. coli* infection (Mann-Whitney two-tailed test, $p=0.0093$). **(f)** Survival assays (n=6, 30mM+ Latrunculin B (Lat B), n=5) were performed in human uroepithelial cells, 5637 after prior treatment with 1µM of Lat B for 1 h prior to 24 h treatment of glucose and estradiol (E2), followed by *E. coli* infection (One-way ANOVA, multiple comparison, $p≤0.05$, $p≤0.01$ respectively). *In vitro* experiments were performed in duplicate or triplicate with at least 3 independent experiments and presented as mean + SEM, statistical outliers defined by Grubb's test were excluded. For *in vivo* analysis individual values and median are shown, $*p < 0.05$, $**p < 0.01$ and $****p < 0.0001$. Source data are provided as a source data file.

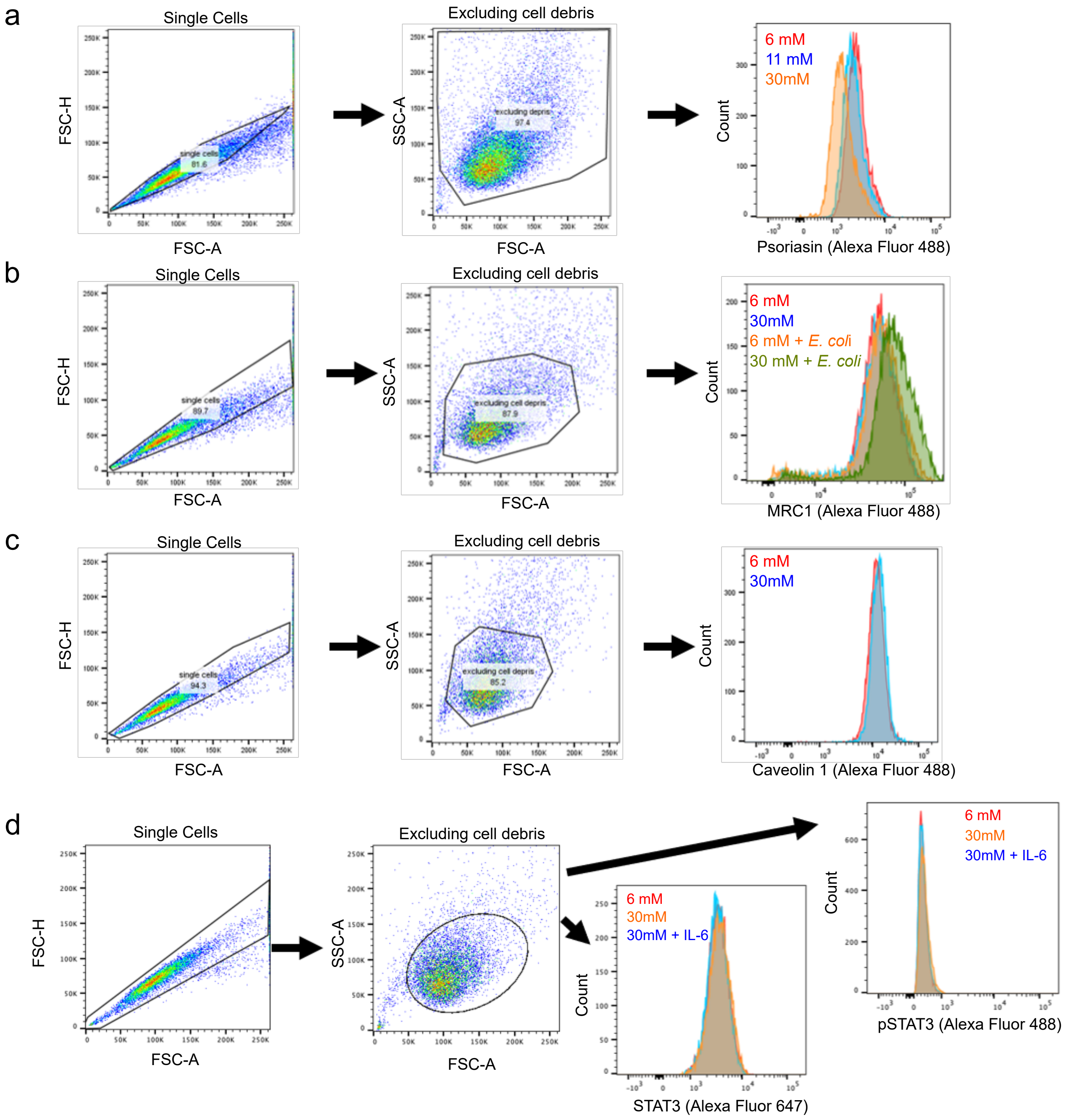

Fig. S5. Gating strategy of flowcytometric data. The gating strategy for **(a)** psoriasin, **(b)** MRC1, **(c)** caveolin 1, **(d)** pSTAT3 and STAT3 used standard FSC and SSC, indicating boundaries between positive and negative cell populations. Representative contour plot, histogram with the fluorochrome for each protein of interest were shown.

Number	Type	Diagnosis	Drugs	Age	BMI	HbA1c	Gender	Duration of diabetes (Years)
1	Control	Hyperthyreosis,<br>status post pulmonary embolus	Protone pump inhibitor,<br>sertralin,<br>glucocorticoid,<br>anticonception pills	36	21.8	NA	F	NA
2	Control	Genital HSV-2<br>most likely asymptomatic	Valaciclovir (intermittent),<br>levonogestrel	35	20.2	NA	F	NA
3	Control	Vasculopathy	No drugs	23	18,0	NA	F	NA
4	Control	Atrial fibrillation,<br>Inflammatory skin disease,<br>arthrosis	Warfarin,<br>angiotensin receptor blocker	74	31.7	NA	M	NA
5	Control	Inflammatory skin disease,<br>Hyperlipedemia,<br>Restless legs	Statin	76	27.2	NA	M	NA
6	Control	Migraine	Beta blocker	24	25.4	NA	M	NA
7	Control	No underlying disease	NA	50	28.7	NA	M	NA
8	Control	No underlying disease	No drugs	66	20.5	NA	M	NA
9	Control	Hypertension	Statin,<br>angiotensin receptor blocker,<br>felodipin,<br>protone pump inhibitor	55	28.3	NA	F	NA
10	Control	NA	No drugs	72	28.1	NA	M	NA
11	Control	Hypertension	Furosemide	77	24.1	NA	F	NA
12	Control	Hypertension,<br>dyslipidemia	Crestor,<br>angiotensin receptor blocker,<br>protone pump inhibitor	66	26.6	NA	F	NA
13	Control	Prolactinoma	Dopamine agonist (bromokriptin)	42	32.5	NA	M	NA
14	Control	No underlying disease	No drugs	44	30.1	NA	F	NA
15	Control	Oesofagus refluxis	Protone pump inhibitor	59	32.5	NA	M	NA
16	Control	Pulmonary obstructive disease (KOL)	No drugs	45	17.1	NA	F	NA
17	Control	No underlying disease	No drugs	49	26.0	NA	M	NA
18	Control	No underlying disease	No drugs	27	20.6	NA	F	NA
19	Control	No underlying disease	No drugs	50	28.9	NA	F	NA
20	Control	No underlying disease	No drugs	25	20.1	NA	M	NA
21	T1D	No other disease	Insulin	38	23.8	64	M	21
22	T1D	No other disease	Insulin	50	32.0	51	M	26
23	T1D	Gastritis	Insulin,<br>esomeprazol	43	22.9	52	F	1
24	T1D	No other disease	Insulin	33	25.8	75	M	29
25	T1D	Colitis,<br>Hypertension	Insulin,<br>enalapril,<br>atorvastatin	43	25.4	70	M	5
26	T1D	No other disease	Insulin,<br>statin	54	27.7	66	M	15

27	T1D	Multiple complications,<br>nephro-, neuro-, retino-pathy	Insulin,<br>statin,<br>amlodipin,<br>candesartan,<br>beta blocker,<br>furosemid	58	25.8	51	M	53
28	T1D	No other disease	Insulin	23	25.1	55	M	9
29	T1D	No other disease	Insulin,<br>naproxen	22	21.5	62	M	10
30	T1D	Chronic pancreatitis (due to ethyl)	Insulin,<br>pancreatic enzymes,<br>vitamin B12	52	23.6	67	M	18
31	T1D	Myalgia NUD	Insulin,<br>glucocorticoid ointment	49	25.0	61	M	19
32	T1D	Multiple complications,<br>(nephro-, neuro-, retino-pathy,)<br>claudication intermittens	Insulin,<br>statin,<br>amlodipin,<br>beta blocker,<br>spironolactone,<br>ACE inhibitor/thiazide,<br>alfuzosin,<br>trombyl	67	33.7	57	M	53
33	T1D	No other disease	Insulin	32	21.2	58	M	11
34	T1D	No other disease	Insulin	38	19.7	38	M	7
35	T1D	Previous recurrent UTI	Insulin,<br>lergigan (mecillinam post coitus)	31	26.4	41	F	17
36	T1D	No other disease	Insulin	46	31.2	73	M	16
37	T1D	Hyperlipidemi	Insulin,<br>statin	52	26.2	50	M	9
38	T2D	Prostate cancer,<br>Hyper lipidimea,<br>retinal vein trombosis	Only diet,<br>statins	81	25.8	43	M	1
39	T2D	Hypertension,<br>Hyperlipidemia,<br>Kidney failure	Insulin,<br>DPP4 inhibitor,<br>statins	44	32.6	61	M	3
40	T2D	Hypertension,<br>Hyperlipidemia,<br>Aorta rupture	Only diet,<br>thyroxin,<br>beta blocker,<br>angiotensin receotor blocker	76	29.1	45	F	1
41	T2D	Hypertension	Sulfonylurea	73	24.6	55	M	4
42	T2D	Hypertension,<br>Dyslipidemia	Metformin,<br>statin,<br>ACE inhibitor,<br>amlodipin,<br>beta blocker	64	30.3	47	M	2
43	T2D	Hypertension,<br>Dyslipidemia	Metformin,<br>insulin,<br>GLP1 analogue	73	28.7	58	M	19

44	T2D	Neuropati,<br>Angina,	Metformin,<br>DPP4 inhibitor,<br>statin,<br>amlodipin,<br>ACE inhibitor,<br>NOAC	67	27.7	50	M	26
45	T2D	Hypertension,<br>Hyperlipidemia,<br>most likely kidney failure due to medication	Metformin,<br>DPP inhibitor,<br>amlodipin,<br>angiotensin receptor blocker,<br>statin,<br>natrium bicarbonate,<br>protone pump inhibitor	69	34.2	51	M	16
46	T2D	Obesity	Only diet	59	32.2	39	M	4
47	T2D	Hyperlipidemia	Metformin,<br>statin	63	26.5	35	M	1
48	T2D	Hypertension,<br>Hyperlipidemia	Metformin,<br>GLP1 analogue,<br>statin	75	33.3	46	M	22
49	T2D	Hypertension,<br>Hyperlipidemia	Insulin,<br>statin,<br>amlodipin,<br>angiotensin receptor blocker,<br>clopidogrel	72	30.5	73	M	15
50	T2D	Hypertension,<br>Dyslipidemia	Pioglitazon,<br>spironolactone,<br>ACE inhibitor,<br>felodipin,<br>angiotensin receptor blocker/thiazide,<br>statin,<br>trombyl	80	34.7	59	M	8
51	T2D	Hypertension,<br>Dyslipidemia	Metformin,<br>sulfonylurea,<br>DPP4 inhibitor,<br>angiotensine receptor blocker,<br>amblodipin,<br>trombyl	76	29.9	55	M	27
52	T2D	Hypertension,<br>Dyslipidemia	Metformin,<br>statin,<br>ACE inhibitor	64	30.2	54	M	2
53	T2D	Hypertension (no drugs),<br>Dyslipidemia	Metformin,<br>statin	80	34.3	42	M	3
54	T2D	Gilberts disease	Metformin,<br>Insulin	61	36.5	53	F	4
55	T2D	Hypertension,<br>Obesity,<br>Hyperlipidemia	Only diet,<br>statin,<br>ACE inhibitor,<br>amlodipin,<br>Fuorosemid	52	30.3	40	M	1
56	T2D	Dyslipidemia	Diet,<br>Statin	64	22.5	42	F	1

Table 1: Clinical information of patients with diabetes and non-diabetic controls used in this study.

**Primers**	
**Gene name**	**Sequence (5'-3')**
Human *S100A7* (Forward)	CACCAGACGTGATGACAA
Human *S100A7* (Reverse)	GGCTATGTCTCCCAGCAA
Mouse *S100a7a* (Forward)	GCTCGTTTAGTGAACCGTCAG
Mouse *S100a7a* (Reverse)	GGAGTCCTCCACTGGTGTGT
Human *IL1B* (Forward)	CACGATGCACCTGTACGATCA
Human *IL1B* (Reverse)	GTTGCTCCATATCCTGTCCCT
Human *IL6* (Forward)	TTCGGTCCAGTTGCCTCTC
Human *IL6* (Reverse)	TGGCATTTGTGGTTGGGTCA
Human *SOCS3* (Forward)	AGCAGCGATGGAATTACCTGGAAC
Human *SOCS3* (Reverse)	TCCAGCCCAATACCTGACACAGAA
Human *AHR* (Forward)	CTGACGCTGAGCCTAAGAAC
Human *AHR* (Reverse)	ACCTACGCCAGTCGCAAG
Human *OCLN* (Forward)	TTTGTGGGACAAGGAACACA
Human *OCLN* (Reverse)	TCATTCACTTTGCCATTGGAT
Mouse *Ocln* (Forward)	CTCCCATCCGAGTTTCAGGT
Mouse *Ocln* (Reverse)	GCTGTCGCCTAAGGAAAGAG
Human *MRC1* (Forward)	TACAAAAAGGACAAACACCAAAACC
Human *MRC1* (Reverse)	TTGTAAATAACCCACCCATCTTCAG
Mouse *Mrc1* (Forward)	GCAAATGGAGCCGTCTGTGC
Mouse *Mrc1* (Reverse)	CTCGTGGATCTCCGTGACAC
Human *CAV1* (Forward)	AGACGAGCTGAGCGAGAAGC
Human *CAV1* (Reverse)	TCGATCTCCTTGGTGTGCG
Mouse *Cav1* (Forward)	ACCGTGCATCAAGAGCTTCC
Mouse *Cav1* (Reverse)	TAGACGCGGCTGATGCACT
Human *RHOB* (Forward)	CATTCTGACCACACTTGTACGC
Human *RHOB* (Reverse)	GGTTTCTTTTCCCTCTCCTTGT
Human *ACT B* (Forward)	AAGAGAGGCATCCTCACCCT
Human *ACT B* (Reverse)	TACATCGCTGGGGTGTTG
Mouse *Act b* (Forward)	CTGTCCCTGTATGCCTCTG
Mouse *Act b* (Reverse)	ATGTCACGCACGATTTCC
Human *HPRT* (Forward)	ATGGACAGGACTGAACGTCTTGC
Human *HPRT* (Reverse)	GACACAAACATGATTCAAATCCCTGA
**Probes**	
**Gene name (Human)**	
CAMP	Hs00189038_m1
DEFB1	Hs00608345_m1
DEFB4A	Hs00175474_m1
DEFB103A	Hs00218678_m1
S100A7	Hs00161488_m1
RNASE7	Hs00261482_m1
18s	Hs03003631_g1

Table 2: List of primers and probes used in this study.
