# Supplementary material for: Diabetes downregulates the antimicrobial peptide psoriasin and increases E. coli burden in the urinary bladder
Source: Nat Commun. 2022 Sep 20;13:4983. doi: 10.1038/s41467-022-32636-y (PMC9489794; doi:10.1038/s41467-022-32636-y)
Supplement: Supplementary file 3 — Reporting Summary [file 41467_2022_32636_MOESM3_ESM.pdf]

nature research

Corresponding author(s): Annelie Brauner

Last updated by author(s): Aug 4, 2022

Reporting Summary

Nature Research wishes to improve the reproducibility of the work that we publish. This form provides structure for consistency and transparency in reporting. For further information on Nature Research policies, see Authors & Referees and the Editorial Policy Checklist .

Statistics

For all statistical analyses, confirm that the following items are present in the figure legend, table legend, main text, or Methods section.

n/a	Confirmed	
☐	☒	The exact sample size (*n*) for each experimental group/condition, given as a discrete number and unit of measurement
☐	☒	A statement on whether measurements were taken from distinct samples or whether the same sample was measured repeatedly
☐	☒	The statistical test(s) used AND whether they are one- or two-sided<br>*Only common tests should be described solely by name; describe more complex techniques in the Methods section.*
☒	☐	A description of all covariates tested
☒	☐	A description of any assumptions or corrections, such as tests of normality and adjustment for multiple comparisons
☐	☒	A full description of the statistical parameters including central tendency (e.g. means) or other basic estimates (e.g. regression coefficient) AND variation (e.g. standard deviation) or associated estimates of uncertainty (e.g. confidence intervals)
☐	☒	For null hypothesis testing, the test statistic (e.g. *F*, *t*, *r*) with confidence intervals, effect sizes, degrees of freedom and *P* value noted<br>*Give P values as exact values whenever suitable.*
☒	☐	For Bayesian analysis, information on the choice of priors and Markov chain Monte Carlo settings
☒	☐	For hierarchical and complex designs, identification of the appropriate level for tests and full reporting of outcomes
☒	☐	Estimates of effect sizes (e.g. Cohen's *d*, Pearson's *r*), indicating how they were calculated

Our web collection on statistics for biologists contains articles on many of the points above.

Software and code

Policy information about availability of computer code

Data collection	cDNA synthesis was performed in MJ research, PTC-200 (Pletier thermal gradient cycler).<br>Real-time PCR was performed in Rotor Gene, RG 3000, rotor gene 6 software, version 6.1.<br>Microscopy images were acquired in LSM 700, Leica SP5, Zeiss AxioVert 40 CFL microscopes with 40x and 63x (oil) objectives.<br>Flow-cytometry data were acquired in BD LSRFortessaTM.<br>ELISA readings were taken in EZ 400, microplate reader (Biochrom) using ADAP software, version 2.0.
Data analysis	Quantification of immunofluorescence was performed using Image J Fiji, software ImageJ 1.53b.<br>Flow cytometry data were analyzed using Flowjo, version 10.8.1.<br>Display of dot plots and statistical analyses were carried out using Graphpad Prism software version 5.

For manuscripts utilizing custom algorithms or software that are central to the research but not yet described in published literature, software must be made available to editors/reviewers. We strongly encourage code deposition in a community repository (e.g. GitHub). See the Nature Research guidelines for submitting code & software for further information.

Data

Policy information about availability of data

All manuscripts must include a data availability statement. This statement should provide the following information, where applicable:

- Accession codes, unique identifiers, or web links for publicly available datasets
- A list of figures that have associated raw data
- A description of any restrictions on data availability

Source data are provided as a Source Data file. Our data do not mandate deposition in a public repository. All raw files and other relevant information are stored in the Karolinska Institutet’s Electronic Lab Notebook. As the Karolinska Institutet’s Electronic Lab Notebook is not a public repository, information may be provided from the corresponding author upon reasonable request.

Field-specific reporting

Please select the one below that is the best fit for your research. If you are not sure, read the appropriate sections before making your selection.

- [x] Life sciences
- [ ] Behavioural & social sciences
- [ ] Ecological, evolutionary & environmental sciences

For a reference copy of the document with all sections, see nature.com/documents/nr-reporting-summary-flat.pdf

Life sciences study design

All studies must disclose on these points even when the disclosure is negative.

Sample size	For in vitro experiments, sample size follows common standards of at least three or more independent biological replicate experiments, consistent with publications in the field. No statistical method was used to determine the sample size. For immunofluorescence experiments in human uroepithelial cells, at least 20- 300 cells per samples were analyzed manually. Sample size is mentioned in the figure legends and each data points were shown using dot plot. Statistical analysis was used to determine the statistical significance of obtained results. p values are reported in the figure legends.
Data exclusions	No samples were excluded from human and animal studies. For in vitro analysis using human uroepithelial cells, statistical outliers defined by Grubb's test were excluded.
Replication	All experiments were reproducible and performed independently three or more times. All biological and technical replicates were mentioned in the figure legends.
Randomization	Randomization was not feasible for this study. All samples in the same experiments were treated in the same manner.
Blinding	In microscopy analysis the same settings were used for respective experimental data collection, relative densitometry was analyzed using Image J, with no need for blinding. In other experiments blinding was not possible as data collection and analysis were performed by the same person.

Reporting for specific materials, systems and methods

We require information from authors about some types of materials, experimental systems and methods used in many studies. Here, indicate whether each material, system or method listed is relevant to your study. If you are not sure if a list item applies to your research, read the appropriate section before selecting a response.

Materials & experimental systems

n/a	Involved in the study	
[ ]	[x]	Antibodies
[ ]	[x]	Eukaryotic cell lines
[x]	[ ]	Palaeontology
[ ]	[x]	Animals and other organisms
[ ]	[x]	Human research participants
[ ]	[x]	Clinical data

Methods

n/a	Involved in the study	
[x]	[ ]	ChIP-seq
[ ]	[x]	Flow cytometry
[x]	[ ]	MRI-based neuroimaging

Antibodies

Antibodies used

Psoriasin mouse monclonal (SC-52948, Santa Cruz Biotechnology); IF, Flow:1:200, In vitro antimicrobial assay: 1µg/ml.
IL-1b Rabbit Polyclonal (P420B, Invitrogen); IF: 1:200
IL-6 Rabbit polyclonal (P620, Invitrogen); IF: 1:100
Phospho-Stat3 (Tyr705) (D3A7) XP® Rabbit mAb #9145 (Cell Signaling Technology); Flow: 1:100
Stat3 (124H6) Mouse mAb #9139 (Cell Signaling Technology); Flow:1:100
Occludin mouse monoclonal (SC-133256, Santa Cruz Biotechnology); IF: 1:200
Occludin Rabbit monoclonal (701161, Invitrogen); IF: 1:200
CD206 (MRC1), (MMR) Recombinant Rabbit Monoclonal Antibody (JF0953) (MA5:32498, Invitrogen); IF: 1:100 or 1:200, flow: 1:200
Recombinant Anti-Mannose Receptor antibody [EPR6828(B)] (ab125028); Mouse IF: 1:100
Caveolin-1 mouse monoclonal (SC-53564, Santa Cruz Biotechnology); IF, flow: 1:200
RhoB Recombinant rabbit Polyclonal Antibody (19HCLC); IF: 1:200
Anti-YAP1 Antibody mouse monoclonal (G-6): sc-376830 (Santa Cruz Biotechnology); IF:1:100
Phalloidin–Tetramethylrhodamine B isothiocyanate, P1951 (Sigma); IF:1:350
Anti-c-Myc antibody produced in rabbit, C3956 (Sigma); IF:1:200
E.coli Goat polyclonal (AbD serotech, OBT 0986); IF:1:200

UPIIIa Rabbit polyclonal (SC-33570, Santa Cruz Biotechnology); IF: 1:200
BD Pharmingen™ Purified NA/LE Mouse monoclonal IgG1 κ Isotype Control (In vitro antimicrobial assay: 1μg/ml)
Alexa Fluor™ 488 Phalloidin (Invitrogen, A12379); IF:1:1000
Donkey anti-Rabbit IgG (H+L) Highly Cross-Adsorbed Secondary Antibody, Alexa Fluor™ 488; Flow or IF: 1:400 or 1:600.
Donkey anti-Rabbit IgG (H+L) Highly Cross-Adsorbed Secondary Antibody, Alexa Fluor™ 350, IF: 1:500.
Rabbit anti-Mouse IgG (H+L) Cross-Adsorbed Secondary Antibody, Alexa Fluor™ 488; Flow or IF: 1:400, 1:500 or 1:600.
Chicken anti-Goat IgG (H+L) Cross-Adsorbed Secondary Antibody, Alexa Fluor™ 488; IF: 1:600.
Donkey anti-Rabbit IgG (H+L) Highly Cross-Adsorbed Secondary Antibody, Alexa Fluor™ 594; IF: 1:600.
Rabbit anti-Mouse IgG (H+L) Cross-Adsorbed Secondary Antibody, Alexa Fluor™ 594; IF: 1:600.
Chicken anti-Goat IgG (H+L) Cross-Adsorbed Secondary Antibody, Alexa Fluor™ 594; IF: 1:600.
Goat anti-Mouse IgG (H+L) Cross-Adsorbed Secondary Antibody, Alexa Fluor™ 647; Flow or IF: 1:400 or 1:600.
Goat anti-Rabbit IgG (H+L) Cross-Adsorbed Secondary Antibody, Alexa Fluor™ 647; IF: 1:600.

Validation

https://www.labome.com/product/Santa-Cruz-Biotechnology/sc-52948.html (This antibody also worked very well for flow and IF of mouse paraffinsection in 1:200 dilutions)
https://www.thermofisher.com/antibody/product/IL-1-beta-Antibody-Polyclonal/P420B
https://www.thermofisher.com/antibody/product/P620.
https://www.cellsignal.com/products/primary-antibodies/phospho-stat3-tyr705-d3a7-xp-rabbit-mab/9145
https://www.cellsignal.com/products/primary-antibodies/stat3-124h6-mouse-mab/9139
https://www.scbt.com/p/occludin-antibody-e-5
https://www.thermofisher.com/antibody/product/Occludin-Antibody-clone-6H10L9-Recombinant-Monoclonal/701161
https://www.thermofisher.com/antibody/product/CD206-MMR-Antibody-clone-JF0953-Recombinant-Monoclonal/MA5-3249
https://www.abcam.com/mannose-receptor-antibody-epr6828b-ab125028.html
https://www.scbt.com/p/caveolin-1-antibody-7c8?requestFrom=search
https://www.thermofisher.com/antibody/product/RhoB-Antibody-clone-19HCLC-Recombinant-Polyclonal/711274
https://www.scbt.com/p/yap-antibody-g-6?gclid=EAIaIQobChMI1cmNjL6F-AIVmgWiAx345weWEAAYASAAEgK9GvD_BwE
https://www.sigmaaldrich.com/SE/en/product/sigma/p1951
gclid=CjwKCAjws8yUBhA1EiwAi_tpEY7g_e6gQjYFRqwLE4qiTVREMZVuke2IomaDwXTy13SinlK39gpumRoCRUcQAvD_BwE
https://www.sigmaaldrich.com/SE/en/product/sigma/c3956
https://www.bio-rad-antibodies.com/polyclonal/bacterial-escherichia-coli-antibody-obt0986.html?f=purified
https://www.citeab.com/antibodies/835179-sc-33570-upiiia-antibody-h-180
https://www.citeab.com/antibodies/10162065-553447-bd-pharmingen-purified-na-le-mouse-igg1-i
https://www.thermofisher.com/order/catalog/product/A12379
https://www.thermofisher.com/antibody/product/Donkey-anti-Rabbit-IgG-H-L-Highly-Cross-Adsorbed-Secondary-Antibody-Polyclonal/A-21206
https://www.thermofisher.com/antibody/product/Donkey-anti-Rabbit-IgG-H-L-Highly-Cross-Adsorbed-Secondary-Antibody-Polyclonal/A10039
https://www.thermofisher.com/antibody/product/Rabbit-anti-Mouse-IgG-H-L-Cross-Adsorbed-Secondary-Antibody-Polyclonal/A-11059
https://www.thermofisher.com/antibody/product/Chicken-anti-Goat-IgG-H-L-Cross-Adsorbed-Secondary-Antibody-Polyclonal/A-21467
https://www.thermofisher.com/antibody/product/Donkey-anti-Rabbit-IgG-H-L-Highly-Cross-Adsorbed-Secondary-Antibody-Polyclonal/A-21207
https://www.thermofisher.com/antibody/product/Rabbit-anti-Mouse-IgG-H-L-Cross-Adsorbed-Secondary-Antibody-Polyclonal/A-11062
https://www.thermofisher.com/antibody/product/Chicken-anti-Goat-IgG-H-L-Cross-Adsorbed-Secondary-Antibody-Polyclonal/A-21468
https://www.thermofisher.com/antibody/product/Goat-anti-Mouse-IgG-H-L-Cross-Adsorbed-Secondary-Antibody-Polyclonal/A-21235
https://www.thermofisher.com/antibody/product/Goat-anti-Rabbit-IgG-H-L-Cross-Adsorbed-Secondary-Antibody-Polyclonal/A-21244

Eukaryotic cell lines

Policy information about cell lines

Cell line source(s)	Telomerase-immortalized human uroepithelial cells, TERT-NHUC (kindly provided by M. A. Knowles, Leeds, UK) (https://pubmed.ncbi.nlm.nih.gov/23785036/). Human uroepithelial cells, 5637 (HTB-9, American Type Culture Collection) (https://pubmed.ncbi.nlm.nih.gov/23785036/).
Authentication	Standard cell lines were employed and showed the expected morphology and growth.
Mycoplasma contamination	All cell lines tested negative for Mycoplasma.
Commonly misidentified lines (See ICLAC register)	No commonly misidentified cell lines were used in the study.

Animals and other organisms

Policy information about studies involving animals; ARRIVE guidelines recommended for reporting animal research

Laboratory animals	Eight-week-old female db/db (BKS (D)-Leprdb/JOrlRj) with type 2 diabetes (median blood glucose: 17.1 mmol/l) and wildtype C57BL/6j mice (median blood glucose 7.1 mmol/l) were obtained from Janvier Laboratories. All mice were kept in a specific pathogen free facility in individual ventilated cages with aspen bedding housing 4 mice per cage. A 12 h light, 12 h dark cycle in ambient room temperature and humidity was maintained, with food and water ad libitum. At week 10, infection was performed. Water was withdrawn 4 h prior to bacterial inoculation, after which water was returned.
Wild animals	No wild animals were used in the study.
Field-collected samples	This study did not involve samples collected from the field.
Ethics oversight	Mice experiments were approved by the Northern Stockholm Animal Ethics Committee, and experiments were carried out according to the guidelines of the Federation of Laboratory Animal Science Association and in compliance with the Committee's requirements.<br>N-177/14 (AB) Studier av cystit och akut njurbäckeninflammation för uppkomsten av njurskada samt möjligheter att förebygga sådan skada, (Amendment 10370-2018).

Note that full information on the approval of the study protocol must also be provided in the manuscript.

Human research participants

Policy information about studies involving human research participants

Population characteristics	All included individuals fulfilled the strict defined criteria for respective diabetes and control group. Detailed clinical information of patients with diabetes and controls is given in supplementary table 1.
Recruitment	Individuals were recruited from a pool of persons who fulfilled the strict defined criteria of the specific diabetes group or controls to be investigated. There was no known self selection bias or biases present.
Ethics oversight	The study was approved by the Regional Ethics Committee, Stockholm and performed in accordance with the Helsinki Declaration. Informed consent was obtained from all patients and volunteers participating in the study.<br><br>1) 2010/723-31/2 (amendment 2018/603-32) (AB) Urine samples from patients with diabetes and nondiabetic controls.<br>2) 96:300 (CGÖ) Hyperglycemic clamp in prediabetic individuals.<br>3) 2009/623-32 (TN) Hyperinsulinemic clamp.<br>4) 2013/1618-31/3 (amendment 2014/1500-32) (KB) Serum samples from nondiabetic control and Type 2 diabetes.<br>5) 2008/1804-31 (amendment: 2017/477-32) (ABr) Serum samples from Type 1 diabetes.

Note that full information on the approval of the study protocol must also be provided in the manuscript.

Clinical data

Policy information about clinical studies

All manuscripts should comply with the ICMJEguidelines for publication of clinical research and a completedCONSORT checklist must be included with all submissions.

Clinical trial registration	Not applicable
Study protocol	Not applicable
Data collection	HbA1c, diagnosis, duration of disease, relevant pharmaceutical drugs used, age, gender and BMI were included.
Outcomes	To compare the possible differences between individuals with diabetes and non diabetes controls.

Flow Cytometry

Plots

Confirm that:

- [x] The axis labels state the marker and fluorochrome used (e.g. CD4-FITC).
- [x] The axis scales are clearly visible. Include numbers along axes only for bottom left plot of group (a 'group' is an analysis of identical markers).
- [x] All plots are contour plots with outliers or pseudocolor plots.
- [x] A numerical value for number of cells or percentage (with statistics) is provided.

Methodology

Sample preparation	TERT-NHUC cells were harvested after 36 h glucose treatment. For MRC1, cells were further infected for 2 h, centrifuged at 350 g for 3 mins at room temperature (RT). 1ml of 4% PFA in PBS (Fisher Scientific) was added to the cell pellet. Cells were incubated at RT for 15 mins, centrifuged and 1ml of 0.1% Triton-X-100 in PBS (PBST) was added and incubated in RT for 10 mins. Thereafter cells were blocked with 5% BSA for 30 mins, stained with primary antibody in 1:1 ratio of 200µl of 1 X PBST and 5% BSA for 30 mins at RT. Primary antibodies used are rabbit anti pSTAT-3 (1:100), mouse anti STAT-3 (1:00) (Cell Signaling Technologies), mouse anti psoriasin (1:200), mouse anti occludin (1:200), mouse anti caveolin-1 (1:200), (Santa Cruz Biotechnology) and rabbit anti mannose receptor c-type 1 (1:200), (Invitrogen). After primary antibody staining, cells were washed with 1 PBS with 1% BSA and further incubated with respective secondary Alexa flour 488 (1:600, Invitrogen) or Alexa flour 647 (1:400, Invitrogen) antibodies in 1:1 ratio of 200µl of 1 X PBST and 5% BSA for 25 mins in dark at RT. Finally, cells were dissolved in PBS and data acquired.
Instrument	BD LSRFortessaTM.
Software	FlowJo, version 10.8.1.
Cell population abundance	The experiment was performed in a cell line with homogeneous cells therefore sorting was not applicable.
Gating strategy	Gating strategy was used using standard FSC and SSC, indicating boundries between positive and negative cell population.

☒ Tick this box to confirm that a figure exemplifying the gating strategy is provided in the Supplementary Information.
